# Being While Doing: An Inductive Model of Mindfulness at Work

**DOI:** 10.3389/fpsyg.2016.02060

**Published:** 2017-02-21

**Authors:** Christopher J. Lyddy, Darren J. Good

**Affiliations:** ^1^Department of Management, School of Business, Providence CollegeProvidence, RI, USA; ^2^Department of Applied Behavioral Science, Graziadio School of Business and Management, Pepperdine UniversityMalibu, CA, USA

**Keywords:** mindfulness, meditation, workplace, organizational psychology, cognition, qualitative research, management, contemplative management

## Abstract

Mindfulness at work has drawn growing interest as empirical evidence increasingly supports its positive workplace impacts. Yet theory also suggests that mindfulness is a cognitive mode of “Being” that may be incompatible with the cognitive mode of “Doing” that undergirds workplace functioning. Therefore, mindfulness at work has been theorized as “being while doing,” but little is known regarding how people experience these two modes in combination, nor the influences or outcomes of this interaction. Drawing on a sample of 39 semi-structured interviews, this study explores how professionals experience being mindful at work. The relationship between Being and Doing modes demonstrated changing compatibility across individuals and experience, with two basic types of experiences and three types of transitions. We labeled experiences when informants were unable to activate Being mode while engaging Doing mode as Entanglement, and those when informants reported simultaneous co-activation of Being and Doing modes as Disentanglement. This combination was a valuable resource for offsetting important limitations of the typical reliance on the Doing cognitive mode. Overall our results have yielded an inductive model of mindfulness at work, with the core experience, outcomes, and antecedent factors unified into one system that may inform future research and practice.

*We did a full hour … of [mindfulness] training… My pager went off like three times. … He's telling us to meditate, and everyone's pager was just beeping. It was not very conducive to meditating*.

*–medical resident*

## Introduction

In the simplest terms, mindfulness is being present, while the purpose of doing work is to achieve future goals. Despite these obvious differences, there is growing interest in workplace mindfulness training to reduce stress and improve performance. Yet the doctor's quote above illustrates the almost-comical challenges with becoming mindful while working. Evidence increasingly suggests that mindfulness can benefit individuals at work without first considering how being mindful fits into organizations oriented toward continuous activity. To optimize training and research around mindfulness at work, we first need to understand if people can be mindful while working. If they can, what is the fundamental nature of this experience?

Mindfulness has been defined as present-centered attention and awareness (Kabat-Zinn, 1990; Brown and Ryan, 2003; Quaglia et al., 2015), and offers broad and significant benefits for individuals at work (Good et al., 2016)^1^. In this definition, awareness involves diffuse consciousness of all facets of an individual's stream of experience, whereas attention reflects a narrow slice selected from that total experience. Impacts span the basic domains of human functioning, including attention, cognition, emotion, behavior, and physiology, which then affect multiple classes of workplace outcomes, including performance, relationships, and well-being. Substantial evidence asserts that mindfulness is a “parsimonious intervention” benefitting the spectrum of individual workplace functioning (Good et al., 2016, p. 134).

Yet these claims gloss over potential dissonance between mindfulness and traditional theories of managerial and organizational cognition (MOC). These theories assume working individuals function in a goal-directed manner (e.g., Walsh, 1995), while being mindful implies numerous cognitive properties potentially disruptive to such models (Weick and Putnam, 2006). As just one example, being present superficially seems antithetical to planning future actions. Therefore, mindfulness and goal-directed cognition have been labeled as the cognitive modes of Being and Doing (Williams, 2008; Kabat-Zinn, 2013; Segal et al., 2013)^2^. Whether and how these two distinct modes interact remains empirically unstudied in the workplace.

Juxtaposing these distinct modes and their properties in Table 1 reveals how dissonant these appear, and raises questions about their mutual co-existence. Mindfulness involves the quality of present-moment consciousness, comprising awareness of reality that is unfiltered by concepts. Experiences are accepted rather than evaluated as good or bad from the self-perspective; free of goals; and released from habitual interpretation, permitting intentional engagement (Weick and Putnam, 2006; Brown et al., 2007; Williams, 2008). In contrast, managerial action generally requires goal-directed cognition enabled by different cognitive properties. These include past and future focus; perception of reality filtered by concepts, interpreted through narratives, and evaluated with respect to the self; and automatic thoughts driving habitual actions (Walsh, 1995; Ashforth et al., 2008).

**Table 1:** **Comparison of Being and Doing modes**.

**Being Mode(Mindfulness)**	**Property**	**Doing Mode (Goal-Directed Cognition)**
**COGNITIVE MODE COMPARISON**		
Present	Temporal Focus	Past and future
Non-conceptual	Perception	Conceptual
Direct experience	Locus of reality	Narratives
Acceptance	Judgment	Evaluation
Self-quieted	Self	Self-centered
Goalless	Goals	Goal-directed
Intentional	Agency	Automatic

Scholars have offered widely divergent views of being mindful for workplace functioning, ranging from complementary to antithetical, and valuable to detrimental (see Table 2). Such ambiguity blocks clear understanding of this construct's organizational integration and impacts. We believe a central gap in this debate is an empirical understanding of how the cognitive modes of Being and Doing interact. Most broadly, can individuals sustain both Being and Doing cognitive modes at work, and if so, how? Subsumed within this exploration lie other key questions, such as do Being and Doing modes mutually support or undermine each other? What are the key antecedents and outcomes of this interaction? Understanding this relationship could elucidate the promise and difficulties of mindfulness at work.

**Table 2:** **Theoretical perspectives on Being and Doing**.

**Relationship**	**Author**	**Quote**
Incompatible	Weick and Putnam, 2006, p. 281 Kabat-Zinn, 2013, p. xvii Brown and Ryan, 2003, pp. 822–823	“Attempts to increase mindfulness in an organizational context are complicated, because organizations are established, held together, and made effective largely by means of concepts. … Conceptual reality is necessary for day-to-day individual and organizational functioning.” “Each of us gets the same 24 h a day… we fill up those hours with so much *doing* that we scarcely have time for *being*.” [Emphasis in original.] “Mindfulness can be considered an enhanced attention to and awareness of … present reality. … This is … contrasted with consciousness that is blunted. … For example, rumination, absorption in the past, … fantasies and anxieties about the future, … awareness or attention … divided, … when individuals behave compulsively or automatically. … Mindlessness, … the relative absence of mindfulness, … [is] these forms of consciousness [that] serve as concrete counterpoints to mindful presence.”
Contingent	Levinthal and Rerup, 2006, pp. 87–88 Dane, 2011: p. 1010 Good et al., 2016, p. 131	“Mindful moments are important if the contexts in which you operate are dynamic.…In less dynamic contexts, … the economies of mindlessness are more appropriate. Mindfulness takes effort and cost; mindlessness in the form of routine can be cost-efficient.” “Mindfulness is … a state of consciousness that may either foster or inhibit task performance.” “Mindful presence in a stressful situation might evoke lower task performance.”
Complementary	Weick and Putnam, 2006, p 281 Brown et al., 2007, p. 213 Good et al., 2016, p. 134	“The most direct way to forestall conceptual moves that mislead is through mindfulness meditation. … Benefits … relevant to organizations, … [are] greater awareness, clearer thinking and better decisions.” “Mindfulness is not … antithetical to thought, but rather fosters a different relationship to it. … [Mindful] people have … the ability to observe the contents of consciousness, including thoughts. … Disentanglement of consciousness from cognitive content may allow thought to be used with greater effectiveness and precision.” “Mindfulness appears to have broad effects on individual functioning, … beneficially influencing many variables.”

The potential ideal for mindfulness at work has been theorized as “being while doing” (Good et al., 2016, p. 132). Despite the growing literature on mindfulness at work, there has been minimal exploration of the relationship of mindfulness and goal-directed cognition. To clarify this relationship, we investigated the experience of working individuals who have tried to integrate mindfulness into their professional work. By reviewing mindfulness and organizational literature, we first bring together disparate views on the relationship between Being and Doing cognitive modes. Our analysis of interviews suggests the relationship between Being and Doing varies significantly and substantively influences workplace outcomes. We close with implications for future research and practice.

### Working mindfully: conceptualizing being and doing

Imagining a monk conducting high-stakes negotiation or stock trading seems almost absurd. Working mindfully evokes such strange juxtapositions, implying calm striving toward goals, and being present amidst thoughts of past and future. The increasing interest in mindfulness at work suggests that this combination may act as a resource offering significant benefits—if only individuals can activate both modes of Being and Doing. Williams (2008) theorizes mindfulness and goal-directed cognition as involving mutually exclusive sets of cognitive properties. They serve different purposes, facilitating different ways of processing and engaging the world. Therefore, he labels them respectively as cognitive modes of Being and Doing.

#### Being mode

Mindfulness as defined above, is available continuously, and can be conceptualized as the momentary state and dispositional tendency of being present (Brown et al., 2007). This quality can be increased through practices involving present-moment focus like meditation and yoga (Eberth and Sedlmeier, 2012). Ultimately, mindfulness involves simply Being with whatever manifests in the present, consistently attending to and accepting the raw stream of experiences (Brown et al., 2007; Good et al., 2016). Mindful individuals exhibit a quieted sense of self, which becomes less central to perception, experience, and behavior (Vago and Silbersweig, 2012). While they may conceptually interpret their experience, they maintain psychological distance (Hülsheger et al., 2014) from these interpretations, and view them as mental content rather than reality itself (Weick and Putnam, 2006). They are less prone to the streams of automatic thought that characterize and govern much of human existence (Brewer et al., 2011), permitting greater intentionality in directing attention, thought, and behavior (Levesque and Brown, 2007; Williams, 2008; Elwafi et al., 2013; Good et al., 2016).

Growing evidence shows that mindfulness broadly and beneficially impacts how individuals function at work (for an extensive review of these impacts, see Good et al., 2016). As this diverse array of benefits appear attainable via practices like meditation, leading organizations like Google, the U.S. Marines, and the Mayo Clinic increasingly offer mindfulness training (Jha et al., 2010; Tan, 2012; West et al., 2014). The nature and implications of this combination are only now drawing interest. Exemplifying these potential impacts, Beckman et al. (2012, p. 3) quote a doctor reporting that mindfulness training led to “thinking more clearly, speaking more honestly, that definitely leaks out into your work, … to have … a … more honest interaction with people.”

#### Doing mode

In contrast, workplace functioning relies upon the Doing mode (e.g., Walsh, 1995; Lord et al., 2010). Organizations produce goods and services, compelling their members to continuously act in a goal-directed manner (March and Simon, 1958; Locke and Latham, 2002). This requires the recall and anticipation of the past and future, with ongoing evaluation of whether present-moment reality is trending toward or falling short of goal realization (Lord et al., 2010). Because the world is complex and uncertain, individuals simplify perception, interpretation, and prediction through use of concepts (Daft and Weick, 1984; Weick, 1995). Such ongoing demands elicit continual and automatic mental activity (Bargh and Chartrand, 1999), particularly a self-concept essential for simulating and evaluating future realities (Baumeister, 1998) that guides present behavior (Markus and Wurf, 1987; Bandura, 1991). These conceptual overlays on experience can appear like reality, leading to Weick's (1979, p. 250) warning that “the map does become the territory.”

#### Theorizing the relationship of being and doing

**Figure 1:**
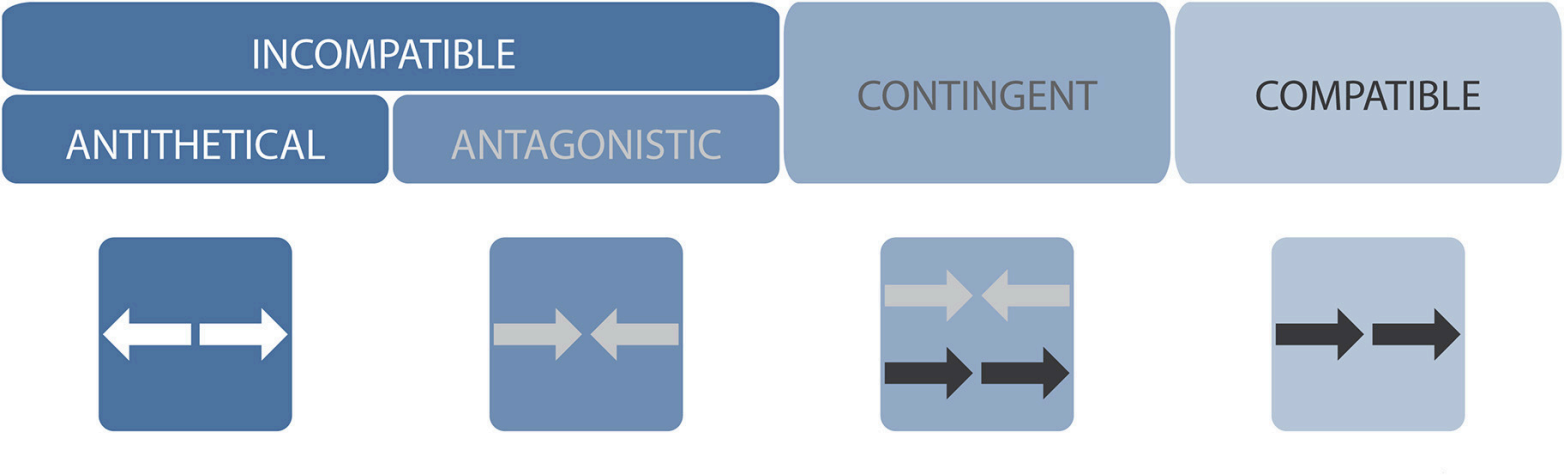
**Depiction of theorized relationships between Being and Doing**.

Theories conceptualize the relationship between Being and Doing as ranging from antithetical to complementary (Table 2). We identify and sequentially describe three types of theories of this relationship: Incompatible, Compatible, and Contingent (see Figure 1). Because the overall relationship of Being and Doing has not been empirically studied at work, this provides a launching point for our qualitative investigation.

##### Incompatible

Some psychological and organizational scholars theorize Being and Doing modes as broadly incompatible, either as antithetical or antagonistic. At the extreme, these two modes may be mutually exclusive across a range of dimensions (e.g., Weick and Putnam, 2006; Williams, 2008), as Being mode involves relative deactivation of cognitive processes integral to work. For example, Weick and Putnam (2006, p. 281) argue that “organizations are established, held together, and made effective largely by means of concepts,” which support planning and coordination (Weick and Sutcliffe, 2006). Therefore, the de-emphasis of conceptual processing in Being mode seems plausibly contradictory to effective organizational functioning (Weick and Putnam, 2006).

Other theories propose that Being and Doing modes have an antagonistic relationship. Being mode may be inversely related to Doing mode properties vital for effective functioning. Mindfulness may correspond with decreased activation in brain regions associated with integral aspects of Doing mode (e.g., Walsh, 1995; Ashforth et al., 2008), like thoughts of narratives, the self, and past and future (Brewer et al., 2011; Vago and Silbersweig, 2012; Tang et al., 2015). For example, automaticity is an aspect of Doing mode (Ashforth and Fried, 1988; Williams, 2008), but mindfulness quells automatic functioning (Brown and Ryan, 2003) and might therefore interfere with executing routine activities (Levinthal and Rerup, 2006).

##### Compatible

Being mode may also be compatible with Doing mode by providing an array of resources benefitting individual workplace functioning (Good et al., 2016). For example, mindfulness training bolsters attention, consequently improving cognitive performance (Mrazek et al., 2013). Given that mindfulness benefits a broad spectrum of basic human functioning, including attention, cognition, emotion, and behavior, it may beneficially influence a wide range of organizational activities reliant upon Doing mode.

##### Contingent

Between these views of Being and Doing modes as incompatible or compatible, a third perspective views mindfulness' value for work as situationally contingent (Dane, 2011). Mindfulness may involve elevated consumption of resources essential to Doing mode (e.g., attention), which may be helpful in some situations but detrimental in others. This logic of contingent value of mindfulness may apply broadly. For example, mindfulness may suspend evaluative judgment and old concepts, which could foster insight and creativity (e.g., Ding et al., 2015), but undermine routinized decision-making. This implies the contingent incompatibility and compatibility of Being and Doing.

These various models capture possible interrelationships between Being and Doing but have emerged without empirical data. This study aims to begin filling this gap. We now turn toward describing the methodology and findings.

## Methods

### Data collection

We interviewed working professionals about how their experiences of mindfulness at work. Our sample included informants with mindfulness experience ranging from brief training programs to decades of meditation practice, Participants completed semi-structured interviews of 30–150 min. When possible, interviews were conducted in person, but logistical difficulties led to some interviews being conducted remotely. Forty three participants were recruited through public and organizational mindfulness groups, direct solicitation, and key contact referrals. Before analysis, four interviews were excluded due to insufficient professional activity or limited understanding of mindfulness. Of the 39 analyzed interviews, 16 participants were male, 23 were female. Ages ranged from early twenties to about 70 years old. Participants had varying mindfulness backgrounds, including contemplative traditions, number of years spent meditating, and types of meditative practices conducted. They also filled diverse occupations, such as doctors, therapists, managers, lawyers, analysts and entrepreneurs.

### Data analysis

Interviews yielded narrative vignettes of how individuals experienced their own workplace functioning while mindful and not mindful (e.g., Riessman, 1993). We analyzed these vignettes through a grounded theory approach (Corbin and Strauss, 1990), clarifying the core facets of the experience of mindfulness at work, as well as antecedents and outcomes.

Our analytical process comprised three stages. The first stage centered around the experience of mindfulness at work. We identified narrative segments in which informants reported being either mindful or unmindful while working. While the data are too lengthy and rich to convey more than select examples here, we offer edited vignettes throughout the paper that convey these focal experiences^3^. We open-coded a subset of the vignettes from 6 interview transcripts, relying on constant comparison among text fragments (Glaser and Strauss, 1967; Suddaby, 2006). Themes emerged regarding the experiences of being mindful and not mindful. We merged content codes into analytical themes like Disentanglement, which were then subsumed under the category of experiences of mindfulness at work (see Table 3).

**Table 3:** **Categories, Themes, and Codes**.

**Categories**	**Analytical themes**	**Content codes**	
Experiences of mindfulness at work	Entanglement: Doing mode prevents Being mode	*Doing mode*	
		Automatic and persistent thinking	
		Temporal focus: Past or future	
		Belief in thoughts	
		Self-centered	
		Judgmental evaluation	
	Disentanglement: Distinct Being and Doing modes co-exist	*Doing mode*	*Being mode*
		Automatic and persistent thinking	Mental quiet
		Temporal focus: Past or future	Temporal focus: Present
		Belief in thoughts	Disbelief in thoughts
		Self-centered	Not self-centered
		Judgmental evaluation	Acceptance
	Transitions: Between Entanglement and Disentanglement	Disentanglement to Entanglement: Became unmindful	
		Entanglement to Disentanglement: Became mindful	
		Continuity of Mindfulness: Remained mindful	
Outcomes of mindfulness at work	Feeling poorly	Stressed, negative emotion, physical maladies^*^	
	Functioning poorly	Ineffective task performance, decision making, and social interaction^*^	
	Feeling well	Calm, clear, spacious, interpersonal connection^*^	
	Functioning well	Effective task performance, decision making, and social interaction^*^	
Antecedents of mindfulness at work	Situational	Attentional, emotional, and task demands	
	Behavioral	Recency of meditation, cumulative meditation practice	
	Individual	Practical mindfulness self-efficacy	

* *While we present content codes here for outcomes, we do not describe these codes in the manuscript for two reasons. First, specific outcomes reported closely reflected previously published research. Second, we chose to collapse rich description of the outcomes from mindfulness into analytical themes to facilitate general theorizing of the experience of mindfulness at work and related outcomes*.

As an example of one core aspect of mindfulness at work, informants repeatedly described experiencing two distinct aspects of their mind active simultaneously. They experienced their normal cognitive activity guiding their workplace functioning, while also having a distinct aspect of their mind observing this activity calmly with psychological distance. They routinely referred to this observing aspect as “mindfulness,” as well as other closely-related terms, such as “awareness” or “spaciousness.” Within these two types of vignettes, patterns of consistent and interrelated themes continuously emerged, such as self-centeredness of perspective while not mindful.

While these core experiences were theoretically anticipated, the transitions between these experiences were unexpected. In the second stage we coded these transitions as experiences of mindfulness at work. We analyzed transitions through a similar process as the core experience categories of being mindful and unmindful, with an additional focus on capturing their before-and-after nature by noting initial and final experiences. While transitions between being mindful and not mindful at work were consistently clear, two subtypes of continuity experiences occurred: avoiding a transition from being unmindful to mindfulness, and reversing such a transition quickly. We ultimately decided that from the emic perspective of informants, these were effectively similar and aggregated them into the category of “continuity of mindfulness.” We then open-coded these transition experiences.

The third stage involved coding the antecedents and outcomes of these core experiences, which served to interrelate them within the workplace context. As before, first-order codes were merged into second-order themes through constant comparison. Because the outcomes from working mindfully and unmindfully closely reflected published frameworks (e.g., Good et al., 2016) and were not the central focus of the study, we subsequently combined these into broad categories of feeling and functioning well or poorly. This allowed us to focus our analysis and manuscript on the core research questions. Multiple categories of antecedents of mindfulness at work emerged as well and are reported throughout. We excluded antecedents for which there was insufficient data to make strong inferences, such as type of mindfulness training received.

With respect to the core experience of mindfulness at work, we believed we reached theoretical saturation (Glaser and Strauss, 1967), as our analytical categories robustly captured the basic experiences of the diverse sample of interviewees. We are confident that the general categories and patterns regarding antecedents and outcomes are also theoretically robust, but given the study's focus on experience, we may not have achieved full theoretical saturation regarding these themes. The intention of this analytical process was to create an inductive model of mindfulness at work, which we now report.

## Findings

### Overview: the experience of mindfulness at work

**Figure 2:**
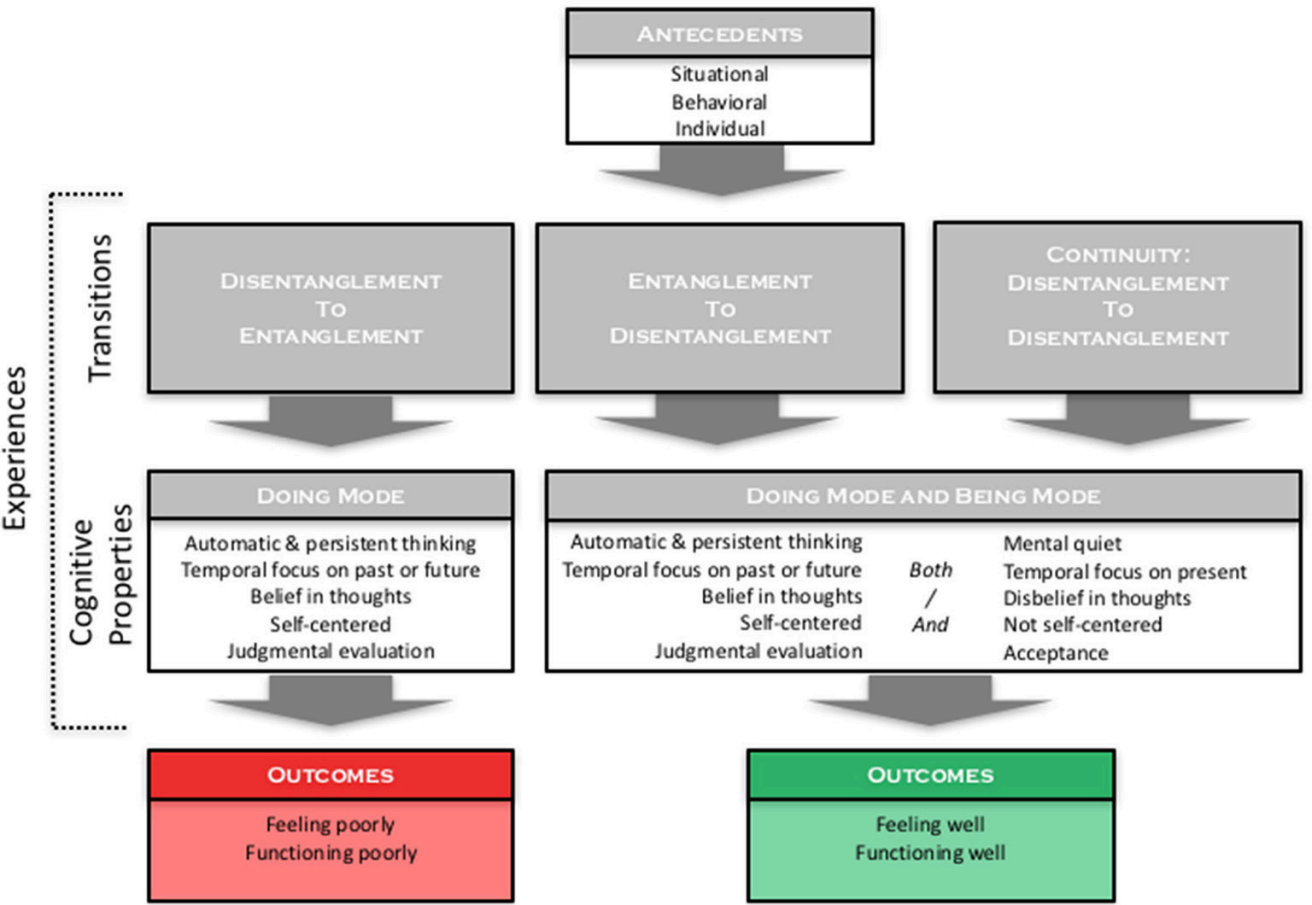
**Inductive model of the experience, outcomes, and antecedents of mindfulness at work**.

Interviewees described how they experienced mindfulness while working. Overall our results have yielded an inductive model of mindfulness at work, with the core experience, outcomes, and antecedent factors unified into one system (see Figure 2). The main conclusion of the model is that individuals can be mindful at work, yet remaining mindful in this environment can be challenging.

Informants reported two main types of experiences. We focus our analysis on reporting the nature and varieties of these experiences, which we broadly categorize as Entanglement and Disentanglement. Entanglement described instances in which informants were unable to activate Being mode while engaging Doing mode. In contrast, Disentanglement involved a reported experience of simultaneous activation of Being and Doing modes. Individuals generally reported both types of experiences, indicating the relationship between Being and Doing fluctuated. This variability was reportedly linked to the occurance of transitions over time in which individuals shifted in three distinct ways: from Disentanglement to Entanglement, from Entanglement to Disentanglement, and from Disentanglement to Disentanglement, the latter representing a Continuity of mindfulness (see Figure 2). They also identified some of the outcomes and antecedent factors of this relationship. Consistent with prior theorizing, informants experienced both benefits and challenges with being mindful at work. Richer description of Entanglement and Disentanglement experiences (see Table 4), with their outcomes and antecedents, follows.

**Table 4:** **Quotes reflecting aspects of Entanglement and Disentanglement**.

**Property**	**Entanglement**	**Disentanglement**
Thinking: Automatic and persistent vs. mental quiet	*When I'm working I … go to autopilot*. *So I ruminate about this stuff, about my image, what people think about me. … There was about 3 h … I can't just leave it, … can't stop thinking*.	*All those things … happened in that spaciousness … I was able to see … what was going on.…And they diffused, the thoughts [and] … the judgments didn't continue*.
Temporal Focus: Past or future vs. present	*I'd prepare in my mind, … and, I'd be living out what I was gonna present and say [in court] … 100 times, or a million times in my head*.	*That's what I mean … being in the present moment, rather than … running off to all these other places that I could be and I have been and I will be*.
Reality of Thoughts: Belief vs. disbelief	You're so embedded in your own thoughts, reactions, judgments, that that's your entire reality. It's happening constantly. *My … state is very much one of believing that thought to be the truth … I can't see anything but that thought*.	It's not the [mental] content that I'm enmeshed with, but rather I'm seeing the process of [thoughts and emotions] arising. I don't have to believe these stories in my head. *Most people … take every thought as … the truth. … If you're mindful … you're looking at [thoughts wondering] ‘Is this really the way it is?’ … [You're] able to … decide which ones are … not correct*.
Self-Perspective: Self-centered vs. not self-centered	My world becomes smaller, my self becomes the center, and … [my] whole perspective … is ‘I’m the most important thing.'	*I am able to … separate myself more from what's happening*.
Judgment: Evaluative vs. accepting	I'm … critical of how well I … support [my clients] … I [was] being so hard on myself, … that can get in the way of me being able to just be in the moment and connect. In a room filled with conflict, [if] I start judging who's right and wrong, I've totally lost my neutrality as a mediator. … Then I'm just like everyone else in the room. How am I helping?	*I was able to … step back and say, … ‘This is what I’m going to do, … that's all I can do.' … Like being [a] vessel. … Being open, absorbing … information … but not reacting to it, … not putting a value on it – positive or negative … accept what's coming in*.

### Mindfulness at work: experience

#### Entanglement: doing prevents being

**Figure 3:**
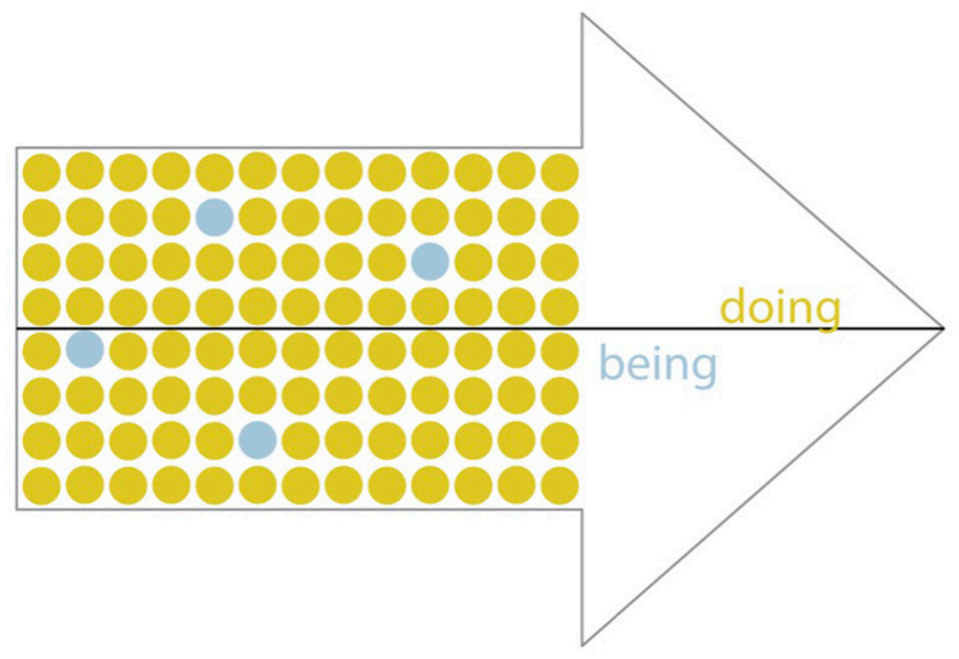
**Depiction of Entanglement**.

Entanglement experiences involved the cognitive mode of Doing preventing activation of Being mode. All informants reported these experiences, when they wished to be mindful but found that Doing mode processing completely filled their mind and prevented any experience of Being mode (see Figures 2, 3). This led informants to describe their experience as being “entangled” in Doing mode (see Table 4) with various labels like “lost in thought,” “caught up,” “carried away,” “engrossed,” and “enmeshed.” This prevented experiencing the neutral, observing awareness of Being mode (a contrasting experience later elaborated), meaning informants were unintentionally and persistently caught in Doing mode.

Informants consistently described their inability to experience Being mode while engaging Doing mode, such as while performing cognitively-demanding tasks. A professor reported that while reading, Doing mode completely filled his awareness, which prevented Being mode activation. He said, “I don't know how you could mindfully read. … You are thinking about … [and] taking in the content of … whatever it is that you're reading. And that occupies my mind. I don't have part of my mind that is watching myself through that reading process.” This reading experience reflected the immersive quality of Doing mode.

Entanglement experiences exhibited core properties including: (1) automatic and persistent thinking; (2) temporal focus on past or future; (3) belief in thoughts; (4) self-centeredness; and (5) judgmental evaluation. These distinct properties were closely interrelated, suggesting they were manifestations of a single underlying experience. For example, judgmental evaluations were often made automatically from the informant's perspective. We now describe the major properties of Entanglement experiences.

##### Automatic and persistent thinking

The Doing mode apparently prevented Being mode activation through incessant, unintended thinking. Informants routinely described their minds continuously cycling through various work-related thoughts. A nurse reported repeatedly thinking, “‘I’ve got this to do, and I've got this to do.' … You're… thinking so much about what else you have to do that you're not concentrating on what it is that you're actually doing.” Similarly, an executive reported that: “When I'm working I … go to autopilot. … It just happens. … To take time to reflect, … I can't do it, … there's too much going on. … From the time that I get in, … I'm going and it's … one thing after the other. … So that's when I move to autopilot.”

The automatic, incessant thinking seemed related to the persistence of Entanglement. Commonly informants found themselves being lured into and then stuck in their mental worlds, despite their intentions. An informant described that: “you're so embedded in your own thoughts, reactions, judgments, that that's your entire reality.” These aspects then not only appeared to dominate functioning, they led to the Doing mode's persistence. As a manager reported, instead of experiences coming and going rapidly, he would be: “enmeshed in … content for a long time, and further more, it would come back later.”

##### Temporal focus: past or future

When Entangled, informants reported temporal focus continuously on the past or future. They frequently focused on prior experiences, particularly unpleasant ones, as a relief worker noted in her response to an uncivil colleague: “The guy was very rude. … I was upset about that all day long. I was ruminating … I [couldn't] just leave it.” Planning future performances also drew their attention, as a criminal defense lawyer described, “What generally gets my mind going is court work. … I'd be living out what I was gonna … say [in court] … a million times in my head. … When you do that, when you actually present [in court], … it's never as good as what's in your head. And you can … actually overdo it, … overprepare. … Before I present [in court], I can get caught up in a different moment, playing it over.” Sometimes past or future focus was instigated by self-concern, as a nurse noted while working during institutional instability: “It was … very stressful. … You're constantly distracted, … ‘Am I gonna have a job tomorrow?’ … It's hard to concentrate on what you're doing, because you're so worried.”

##### Belief in thoughts

With their focus outside of the present, Entangled informants also continually found themselves believing in their own thoughts as the unquestioned truth. A manager exemplified how Entanglement experiences involved fully believing in the mind's content. He described that after recognizing a work-related problem he was responsible for, “the thought comes in, ‘This is a bad situation,… we should have … [caught] this thing with our testing.’… My … state is very much one of believing that thought to be the truth, … I can't see anything but that thought.” Total belief in such narratives led them to ultimately govern behavior. One therapist reported getting into an argument with a client diagnosed with mental health problems:

*I … had a patient who … was … yelling. … Instead of being [mindfully] present and knowing that she was really … irrational, I started to have the conversation with her on the content of what she was talking about, instead of seeing the bigger picture. … She was criticizing me. Instead of being able to sit back and understand where she was coming from or why, I got too caught up in the details*.

Despite acutely understanding that the client was enacting a distorted perspective, the therapist nonetheless was unintentionally drawn into the content of the conversation, reflected in her being temporarily “caught up” in this story.

##### Self-centered

Interviewees also described Entanglement experiences as involving *self-centered* interpretation of reality. An informant exemplified this in saying that, “my world becomes smaller, my self becomes the center, and … [my] whole perspective … is ’I'm the most important thing.”’ The self-centered perspective could strongly shape interpretations to situations and responses, as a mildly critical email provoked an egocentric reaction from a billing manager:

I … get an email, … ‘you did this invoice wrong because the system is different.’ … But the ego-based thought was … ‘that’s because you changed the system after the guy signed the contract.'.…I want to get into it with this guy so bad in that moment! It was nothing to do with the… getting the process right, … or having a common goal, or any of those things will take me out of that particular reactive mode … That reactive mode is consistently ego, it's like, ‘Who do you think you are?’

Often events threatened an informant's well-being or plans, provoking self-concerned threat or disappointment. A financial analyst described his self-centered reaction to internal debates, saying, “I can be very aggressive. … If someone attacked me, … I could go into attack mode also. … They might say: ‘What are your assumptions? … How did you get to that conclusion?’ … I could say, ‘Who the hell are you? I'm the [expert]! You don't … understand the models.’… That is never productive, … that … defensive, angry, …arrogant energy.”

A medical resident reflected this in describing his response to a rejected application for a training fellowship: “I didn't get my fellowship … so it's been … a personal challenge. … I was very, very upset. You have a lot of things going on in your head. … Where am I going to go? … How am I going to tell my parents? All of these things go into play.”

##### Judgmental evaluation

Entanglement also manifested as *judgmental evaluation* of experiences as good or bad. As a therapist continually evaluated if she met her own performance standards, this prevented Being mode activation and hindered her effective functioning:

*I want to … be the best therapist. … I was struggling in my … session with [a client], feeling like I wasn't able to … identify … the issues. … I [was] being so hard on myself, … that can get in the way of me being able to just be in the moment and connect. … I'm having this judgment of how I'm doing, so I might not … pick up on … facial gestures or her affect, … rather than staying emotionally with what's going on for my client … I'm not able to do it as well when I have this judgment*.

A professional mediator reported a similar experience of judgmental evaluation linked to challenges with functioning, who described: “with a client … when I'm judging them, I'm totally ineffective, … I've lost my capacity to function. … In a room filled with conflict, [if] I start judging who's right and wrong, I've totally lost my neutrality. … Then I'm just like everyone else in the room. How am I helping?”

In all, despite intentions to be mindful at work, informants reported that during Entanglement experiences, Doing mode dominated cognition and prevented Being mode activation. The reverse was not reported; no informants experienced Being mode preventing Doing mode activation. Nonetheless most informants also reported the ability to work mindfully, and we now turn to this experience.

#### Disentanglement: being while doing

**Figure 4:**
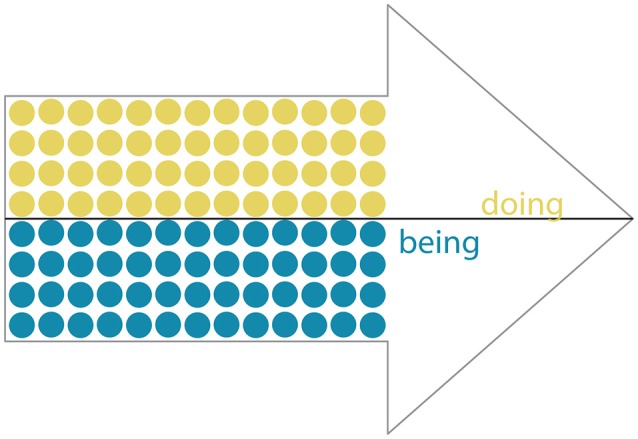
**Depiction of Disentanglement**.

In marked contrast, Disentanglement experiences involved simultaneous co-activation of Being and Doing modes (see Figures 2, 4 and Table 4). We chose this term to contrast with the *in vivo* term “entanglement,” experiences in which Doing mode prevented Being mode activation, and to reflect existing terminology in Brown et al. (2007). During Disentanglement experiences, Being mode was no longer deactivated by Doing mode, but emerged as a second, independent entity leaving the mind “disentangled” with two distinct cognitive modes. Properties consistent with both Being and Doing modes were reported during these experiences. As with Entanglement, these were distinct but mutually interrelated aspects, suggesting they were manifestations of a single underlying experience.

Exemplifying this experience, one manager described being mindful while working: “mindfulness is… a quality of awareness, … of what's happening and the process of what's happening. So you're living the content [of your work or task], but at the same time, fully aware of the process of what's happening.” In this articulation, “process” referred to how mental content emerged within consciousness and shaped workplace interpretation and functioning. Experiences of Disentanglement involved holding the active mindful awareness of Being mode while simultaneously engaging in typical Doing mode processing enabling workplace functioning. The primary experience was of two modes operating as parallel layers, when informants experienced Being and Doing as distinct and separate modes jointly determining their cognitive processing.

Disentanglement experiences fundamentally involved Being while Doing (see Figures 2, 4), as both cognitive modes shaped informants' cognitive processing of their workplace situation. Informants experienced Doing cognition similarly as in Entanglement experiences, filled with automatic thoughts, including self-centered reflections, plans, and evaluations. Unlike Entanglement experiences, this was not the only mode of mind governing their workplace functioning. They also simultaneously experienced Being mode as another aspect of the mind, described as quiet, present-focused, clear, and accepting awareness. Reflecting this state, an introverted nurse described confronting a bullying aide while experiencing the limitations of Doing mode and drawing benefits simultaneously from Being mode. She said, “I'm … a pretty shy … person. … I feel very ungrounded when I feel like that. So being mindful, even if I still feel all anxious, … there's a place that I'm still grounded … [that lets me] be a little more personable, and aware, and not totally lost.” Note that she reported properties of Doing mode impacted her functioning, as her anxiety reflected self-focus and potentially judgmental evaluation, but this was offset by the sense of being “grounded” conferred through Being mode.

Reflecting many descriptions of psychological constructs (e.g., spotlight of attention, stream of consciousness), informants commonly used metaphors to convey experiencing Being and Doing modes as parallel layers of the mind. Popular metaphors included natural substances; a manager described his experience of Being and Doing as “different spaces, it's like having… layers, … like oil and water.”

Informants commonly reported moments when these two modes of Being and Doing were Disentangled. This allowed Being mode to provide different responses to workplace situations than those governed by Doing mode. These properties comprised: (1) mental quiet; (2) temporal focus on the present; (3) disbelief in thoughts, (4) lacking a self-centered perspective, and (5) acceptance rather than judgmental evaluation. Similar to Entanglement, these properties were distinct but regularly co-occurred, suggesting they were manifestations of an underlying state. We now describe these major properties of Disentanglement experiences.

##### Mental quiet

While Doing mode reportedly involved an automatic and incessant stream of thought, Being mode reportedly involved mental “quiet.” Participants described this with various related terms like mental “calm,” “spaciousness,” or “stillness.” A finance manager found this aspect of mindfulness helpful in moments when her job was overwhelming: “I have a lot of people asking me for a lot of information. … Being mindful helps a lot … because it calms your mind even though you're, … going so fast. You're still. There's .… a quietness in the background.”

Exemplifying how Being and Doing modes co-existed while processing workplace situations, a senior manager spoke about an engineer who mismanaged another workers' time. He described the responses manifesting within two layers in his mind: his Doing mode evaluated the situation, a cognitive response manifesting within the “spaciousness” of Being mode. In this case, Being mode appeared to influence the automaticity of Doing mode, dissipating ongoing thoughts. He said:

*I could see the engineer getting all agitated … [and] feel my mind judging, … that he shouldn't have given … tasks that were going to consume that [subordinate's] time, [and judging] … myself for ‘why wasn't I closer to this situation?’ … Those things … happened in that spaciousness. … I was able to see … what was going on … to be only a [sense of] threat, … an awareness, ‘this is what's happening, … there’s no real emergency.'… And they diffused, the thoughts … didn't continue*.

##### Temporal focus: present

Disentanglement implied that while informants might think of past or future through Doing mode, they also maintained simultaneous consciousness of the present through Being mode. An attorney described court days as involving consistent activation of Doing mode, but that Being mode manifested as detached awareness of the present: “a lot of the day you are … in the thinking mind, because you gotta be there. But … you can… still be grounded. … the thinking mind is still active, but you're still present, so … you're aware of the thoughts from that … awareness.”

In the following example, a hospital chaplain described her attempts to remain present-centered with patients despite the chaotic environment pressuring her to recall or anticipate events. She reported: “Here I am, … rather than … my mind in all the different cases that I'm working with, being able to really *be*, [to] feel like I've slowed down. … Rather than running off to the last patient, … somebody's crying on my shoulder. … That's … being in the present moment, rather than … all these other places that I could be, and I have been, and I will be.”

##### Disbelief in thoughts

While Disentangled, informants experienced themselves as viewing the Doing mode's thoughts and stories from the distant and quiet spaciousness of Being mode. Central to this property was seeing the cognitive process of story generation, as one manager reported: “when … mindful… it's not the [mental] content that I'm enmeshed with, but rather I'm seeing the process of [thoughts and emotions] arising.” In turn, this produced skepticism that Doing mode-generated workplace narratives reflected reality. One interviewee reported that because of mindfulness, “I don't have to believe these stories in my head.” An entrepreneur described disbelieving the story almost as a scientific process, saying: “most people … take every thought as … the truth. … If you're mindful, … you're looking at [thoughts wondering] ‘Is this really the way it is?’ … [You're] able to … decide which ones are … not correct.”

As Doing mode stories were often dramatic, this distance fostered engaging work with greater equanimity. This contrasted with Entanglement experiences, when informants were immersed in dramatic narratives of work. As one informant noted, when working mindfully they could instead “see the movie… without becoming an actor” in these stories. A lawyer described perceiving legal adversaries as threatening, which provoked self-centered thoughts of insecurity that could impact his performance. Through mindfulness, he began to doubt the reality of these thoughts. He said: “The 800-pound gorillas of law … [make] you frightened. … When you use mindfulness,. … you can find out where the button they're pushing is wired to,.… and you go … ‘I was insecure. … But now is this real?’ … You take away a lot of the … current that's running through that circuit.”

##### Not self-centered

As Being mode involved a quieted mind, informants no longer adopted the self-centered focus characteristic of Entanglement; rather they saw themselves and their situation from a detached, neutral perspective. An analyst reported that when meditation led him to feel mindful, work seemed easier because: “I'm not so wrapped up in my petty little worries and concerns.” One nurse reported that working amidst her busy and demanding environment, being mindful was: “quieting, you could get rid of a lot of the chatter. … I am able to … separate myself more from what's happening.”

##### Acceptance

Disentanglement experiences included acceptance of workplace experiences and events via Being mode, even as participants also evaluated situations through their Doing mode. One example occurred around goal-directed behavior. A manager reported holding goals and planning actions to realize them, thinking occurring through Doing mode, but also being unattached to outcomes and progress, an aspect of Being mode. He described the experience as having: “little expectation for what's going to happen. I … do something intentional toward a goal, but if that's thwarted along the way … that's just as good as if I'd accomplished [my goal.]”

Individuals reported consistently accepting a wide range of workplace situations, including those that would normally be deemed unpleasant or threatening. One analyst reported this property impacted a difficult interaction with her boss. She said:

*I was able to … say, … ‘This is what I'm going to do, … that's all I can do.’ … If I hadn't been in that frame of mind, I would have been … panicking. … Being open, absorbing … information, … not putting a value on it, positive or negative. Not having it influence or change me. … Just … taking it in. … It doesn't mean that you're not having the emotions … but, … you're not going to … react to it*.

By accepting the situation, she experienced her responses without feeling like they needed to translate to action.

Overall, Disentanglement involved mental quiet, temporal focus on the present, disbelief in thoughts, lack of self-centeredness, and acceptance. While the cognitive properties of Doing mode were also available, activation of Being mode meant those properties no longer dominated the mind. We now turn toward describing the transitions that determined if individuals become Entangled or Disentangled.

#### Transitions between entanglement and disentanglement

Contrasting with prior theory, the relationship of Being and Doing modes was not fixed; instead it appeared to change across individuals and situations. Informants who were Entangled would become Disentangled, and vice versa; both experiences were common (see Figure 2). Typifying the experience of mindful professionals, a nurse asked if she found challenges in being mindful at work replied, “Yeah, all the time. It's not something that once we do it, it's done. … You slip 5,000 times. … Sometimes you'll forget. … Maybe next time … taking that 2 s to … focus on what's going on right at that second.” Some also reported sustained periods of Disentanglement free of Entanglement, which they described as a “continuity of mindfulness.” Because Entanglement and Disentanglement had significant implications for workplace functioning, these transitions impacted workplace behavior and outcomes.

##### Becoming entangled

Individuals who intended to be mindful at work nonetheless frequently reported becoming Entangled, particularly in evocative or distracting situations. As one office administrator described:

*If there's something gripping [or] very compelling that's happening, mindfulness goes way down for me. … [The situation] draws my mind out of itself and into the experience, rather than being able to step back and be aware. … If I'm getting yelled at by my boss, … I'm aware that he's talking to me, and that's it*.

In this situation, Being mode was initially active alongside Doing mode, but the event intensified the Doing mode, absorbing the informant into his own mental content. This then inhibited Being mode, resulting in Entanglement.

##### Becoming disentangled

A reverse process also occurred, as informants who were Entangled became Disentangled. In such instances, they were initially immersed in Doing mode fully and had lost awareness of Being mode. By reconnecting with the Being mode, often by focusing attention in the present through mindfulness practices (e.g., breath meditation), they experienced Disentangling. In these instances, the Being mode re-emerged alongside Doing mode.

Activating Being mode during a situation often dissipated interviewees' undesired automatic reactions. A relief worker coordinating a day's activities found herself reacting at the perceived irresponsibility of a colleague. Left unchecked, this reaction could have provoked an unpleasant interaction. Instead, she meditated, became Disentangled, and resolved the situation more favorably:

*I found myself getting upset, … these thoughts … were taking over, … I assumed that there was going to be an issue. … Instead of losing it, I was able … to … do … meditation, … [and] get to this calm place where I can really see … the steps that I can be taking. … I texted him … ‘Hey, did you get this … and that?’ He was like ‘Sure did!’ … In my mind, I had made it this big thing. … It was never an issue*.

Her initial Doing mode response was to automatically anticipate potential problems and blame them on her colleague. By meditating and becoming Disentangled, she quieted her initial appraisal of blame and reconsidered the situation, ultimately identifying an easy correction and avoiding unnecessary interpersonal conflict.

##### Remaining disentangled: continuity of mindfulness

Participants reported that they sometimes experienced persistent Disentanglement, which they termed a “continuity of mindfulness.” In some cases, this reflected a baseline quality of co-active Being and Doing modes that infused an informant's daily work activities with little effort or intention. Sometimes this could be disturbed, as Doing mode threatened to absorb awareness and induce Entanglement. However, some individuals reported an ability to notice and prevent or rapidly reverse this process of becoming Entangled. In either case, informants remained Disentangled at work independent of their circumstances.

Informants often found that Being mode was active without effort or conscious intention as they engaged their work through Doing mode. One informant described: “I don't see how I would … be mindful and have it separate [from my work]. It's part of my life, part of who I am. … I don't know how you could … not bring it to work.” A manager reported that this Continuity emerged over the years he practiced meditation. He said: “When I started meditating, it was a separate experience. … I would … focus my mind. The rest of the time, …, I just went back to everyday life. … So now when I sit in meditation, it's no different than when I'm not sitting in meditation. … There's a continuity, … it's not a separate thing. … No matter what, … that presence is still there.”

While informants described having this undisturbed Continuity, they often reported averting or reversing Entanglement to maintain their Continuity of mindfulness. Disentangled interviewees kept their sense of self removed from the situation, fostering calm and intentional responses. In one such instance, a therapist described being yelled at aggressively by a patient, which provoked a strong reaction and potential for Entanglement. Activating Being mode allowed her to avoid becoming Entangled, and consequently, to choose a more helpful clinical response. She described that the patient was:

standing up, … talking to me really fast, … loud, and … upset. … Then I notice myself having … a fight or flight reaction. I have to be really aware of that, because they need me to … hold that … reaction, … and to respond to their aggression in the right way. … You're … put into submission … verbally. … I was having a reaction, and I wanted to communicate it to her … because she was totally unaware, … so I just bent over in submission. … She said, ‘Okay,’ … she was done. … It was helpful for me just to be sitting … mindful, just being aware, ‘Oh. This person's standing … above me. They're yelling at me.’

### Mindfulness at work: outcomes

Participants routinely described that Entanglement experiences often coincided with undesirable workplace functioning, issues that could be avoided or corrected through Disentanglement, which coincided with positive workplace functioning. As reported outcomes coincided closely with previously published research (e.g., Good et al., 2016), we avoided lengthy cataloging of outcomes. Instead we collapsed these into broad categories of feeling and functioning poorly and well. Feeling and functioning poorly was linked to Entanglement, while Disentanglement consistently corresponded with feeling and functioning well. We illustrate this pattern to demonstrate the process through which the relationship of Being and Doing shaped workplace outcomes.

#### Entanglement: feeling and functioning poorly

When Entangled, informants only had their Doing mode active, constraining the cognitive properties available for engaging workplace situations. The following examples illustrate some resulting negative implications of this limitation. An entrepreneur described problems arising from automatic evaluative thoughts: “In business, you're in charge of a lot, … and your brain is just … on top of itself, telling you all of these things that you're doing wrong, that you need to be doing. … I didn't know how to quiet all that chatter down. [It was] to the point where it was getting in the way of my creativity and functioning.”

Likewise, a manager described that the automatic and self-centered interpretations of Doing mode could be problematic, instigating her uncivil interaction with her boss:

*Usually my non-mindfulness is … taking the action that I shouldn't have taken, rather than not having taken an action that I should have taken. … The boss said something about something he wanted differently. And he said ‘I'm not trying to get on your case or anything.’ And I said ‘yes you were!’ I should not have said that! … He was right … It was not real cool. … These words just flew out of my mouth that I shouldn't have spoken*.

#### Disentanglement: feeling and functioning well

Disentanglement amidst a workplace situation often helped avert problems that arose through exclusive reliance on Doing mode, and therefore benefitted workplace experience and functioning. After discovering a freezer malfunction causing significant business disruption and financial loss, a small café owner reported that being Disentangled coincided with effectively responding. She recounted:

*Your heart sinks, because you're thinking about the money and the … customers … When something goes terribly wrong … I'm able to pull out of that, [to] go from [thinking] ‘ohmygod ohmygod, all my [food] is all defrosted, … I'm panicking.’ … But then … I just stop and I breathe. … You just stop it all. … You go, ‘What am I gonna do about this? OK, I'm gonna call this person, they're gonna be able to get that.’… Before, I would have been mad at myself for 3 days … but in a couple of hours, I fixed the problem,… and that is what the mindfulness helps me with*.

An initial disruption triggered rapid, automatic thoughts filled with negative evaluations of the situation, which in the past had persisted for some time. As she became mindful through meditation, this aspect of Doing mode subsided. Instead her evaluative thoughts quieted, and with this new mental clarity, she was able to identify reasonable actions to address the problem.

In another incident, the therapist who previously reported self-judgment undermined her pursuit of excellence found activating Being mode helpful. She said:

*Meeting with [a client], … I'm criticizing myself, … ‘you're not being helpful with this client.’ … I was aware, … I recognized that. … It helped me then to be confident. … I proposed a concrete intervention that I think she felt was helpful, whereas before I had been … timid and passive. … Being aware that I was feeling this insecurity … helped me to take the risk. … [Mindfulness supported this by] … being aware of [my insecurity] in the moment*.

Her ongoing internal critique was driven by Doing mode, and the associated timidity left her engaging her client in an ineffectual manner. Becoming aware of her self-evaluation through mindfulness let her engage more confidently, allowing her to take risks and ultimately propose interventions that the client found valuable.

Overall, the stories indicated that Doing mode was essential to work but also imposed a constrained set of cognitive properties that could interfere with working effectively. While Entangled individuals were constrained to the properties of just Doing mode, Disentangled individuals were to use these while also drawing upon the properties conferred via Being mode. This diversity averted downsides of Doing mode, and ultimately enabled more effective functioning.

### Mindfulness at work: antecedents

The fluctuating relationship between Being and Doing modes appeared to be influenced by situational, behavioral, and individual antecedents. We now complete the model by describing the most reported factors influencing the Being-Doing relationship (see Figure 2).

#### Situational antecedents of being mindful at work

Key situational factors impacting the experience of mindfulness at work included attentional, emotional, and task features. These features could interfere with mindfulness by disrupting present-moment focus, or support mindfulness by facilitating attention to the present. A high school teacher reported that he was unable to be mindful in his demanding classroom: “I'd park [at school]… and the next thing I'd remember about being mindful was opening up the door to go home. … High school is such an intense place, … there was so much distraction pulling me away.” Disruptions were often emotional in nature. An analyst reported that “someone will just be rude out of nowhere, and it catches you off-guard, … you're trying to keep it together. … It's when mindfulness is … the hardest to come by.” Task demands provoked automatic thought that often undermined being mindful. This was exemplified by a nurse who described: “There's always a lot of stuff going on here, … [which] makes it hard [to be mindful.] … My brain has … to fight a battle. Should I be paying attention to those 25 things, or should I really just be here?”

Despite these challenges, the workplace context was sometimes favorable for mindfulness. The same teacher reported being mindful was much easier after he moved to a private office imposing less attentional demands. In this environment he believed that: “There's more of an opportunity to maintain continuity of mindfulness during the day. Not that it's 100%. It might be 20% of the day, … but it's more. … Less distraction … means that generally I'm more mindful during the day.”

Some informants found Being mode was essential to performing their jobs, and consequently reported greater ease in being mindful. Informants with this perspective typically had roles emphasizing relational quality, such as therapists and nurses. A therapist found that her ability to function effectively was heavily dependent on being present with her patients. She said, “I could easily see 8–10 patients back-to-back. … It's a lot of trauma, … depression, anxiety. … Literally, if you can't stay present with your patients, … you're screwed. You won't be effective, … you will drown.” Similarly, a nurse found that despite many task demands, relating in the moment to patients was vital to working effectively. She described that, “the most important thing is just to … be here now. … Not constantly jumping to the next thing; not feeling that pressure of ‘I've got all this other stuff to do.”’

One particularly striking example was that of chaplains. These informants were explicitly forbidden from performing typical healthcare activities, like patient diagnosis and treatment. Instead their formal job description was to simply be present with patients and families as they faced disease and suffering. One described how this role virtually required Being mode: “Our training is that you can't fix [people's problems]; you can just be with [them]. And that somehow does offer some release for people. … This training is beating the ‘fix-it’ mentality out of you. … Being very, very careful to be a non-judgmental presence. … That's why I'm drawn to mindfulness, because it intersects so readily with my practice as a chaplain.”

#### Behavioral antecedents of being mindful at work

As expected, a key behavioral antecedent impacting mindfulness at work was the practice of meditation. When completed effectively, this reportedly supported Disentanglement and benefitted Continuity of mindfulness at work, an effect strengthened by recency of practice and cumulative meditation expertise. One nurse described taking a few seconds to integrate meditation into her routine task work, saying that, “when I'm washing my hands, I try to feel the water, so there's some seconds of mindfulness in my daily routine. … I feel more grounded … for a little while.…But … because I do it for like 10 s, … it can't last very long.” These effects could build up with repeated practice, leading one to say, “if I'm meditating for … 5 days … consecutively, I feel a stronger sense of just being happy, … at peace. … There's … a center of contentedness, but the stress is eating away at it the rest of the time. … I have more of that content feeling when I meditate. … It needs that buildup.” While this example reflected practice over a week, cumulative lifetime practice was noted as particularly supportive of Continuity.

#### Individual antecedents of being mindful at work

Among individual factors, informants believed they held varying effectiveness at being or becoming mindful at work, particularly around performing mindfulness practices like meditation. A relief worker described her difficulty meditating near her colleagues, saying: “You're always under the gaze of others from your own mind, … that … never really quite settles down. … I just wasn't really able to focus on meditation.” In this case, the person did not feel capable of becoming present in that context. We labeled this self-perceived degree of effectiveness as *practical mindfulness self-efficacy*.

## Discussion and implications

### Overview of findings

Mindfulness and goal-directed cognition have been termed cognitive modes of Being and Doing (Williams, 2008); therefore mindfulness at work may be theorized as “being while doing” (Good et al., 2016, p. 132). Because the relationship between Being and Doing has been thought to range from Incompatible to Compatible (e.g., Weick and Putnam, 2006), achieving this combination may be both beneficial and challenging. Clarifying these disparate theoretical views motivated the study, to our knowledge the first to empirically explore this topic.

The study contributes to existing theory by showing that Being and Doing modes exhibit a complex relationship that is not fixed (e.g., Williams, 2008; Kabat-Zinn, 2013), but instead fluctuates across individuals and situations. This relationship and associated transitions anchor our inductive model of mindfulness at work, depicted in Figure 2. Our findings affirm that maintaining mindfulness at work is valuable but also challenging, as the workplace context can inhibit mindfulness. We now consider these findings for contributions to research, focusing on the experience of mindfulness at work, along with its outcomes and antecedents. Next we consider how these findings help to situate mindfulness within existing conceptions of MOC. We end with implications for practice.

### Implications for research

Our study was intended to explore the nature of mindfulness at work. The findings suggest that prior research may have sometimes overstated the challenges and understated the complexity of being mindful at work. Informants reported that being mindful at work was possible, but often hard to maintain. Not surprisingly, informants linked their capacity to be mindful while working with its perceived value.

#### Experience of mindfulness at work

Our findings show that Being and Doing are varyingly Incompatible and Compatible, which reflects informants' experiences of Entanglement and Disentanglement (see Figures 2–4).

These interviews suggest two main shifts in theorizing the nature of mindfulness at work. The first is that Disentanglement reflects a hybrid cognitive mode blending properties of Being and Doing. When individuals maintain this state, they enjoy the ability to use cognitive properties from both modes, adding psychological resources for engaging work (e.g., the ability to simultaneously evaluate and accept one's performance), seemingly without imposing limitations (Figure 2).

The second is that Disentanglement appears highly unstable. Individuals struggle to be mindful at work and became Entangled, limiting them to Doing mode's cognitive properties (e.g., automatic thought, evaluation, etc.) to guide their functioning. These findings mirror quantitative evidence demonstrating large fluctuations in state mindfulness within individuals (Hülsheger et al., 2013; Jamieson and Tuckey, 2016). Therefore, mindfulness at work should not be viewed as a static state that has been achieved; rather individuals must continually anchor themselves in the present by managing frequent transitions between Entanglement and Disentanglement.

#### Antecendents of mindfulness at work

The data also suggest that while transitions between Entanglement and Disentanglement are common, not everyone manages these with equal skill. Extending Reb et al.'s (2015) exploration of the organizational antecedents of mindfulness, we found that situational, behavioral, and individual antecedents impact the ability to remain Disentangled. For example, some jobs display greater consistency with Being mode (e.g., hospital chaplains). Likewise, individuals reportedly display a range of perceived ability to enact mindfulness practices at work, which we refered to as *practical mindfulness self-efficacy*. Therefore, dispositional mindfulness and general meditation ability may not fully transfer to the workplace—perhaps making these too imprecise for accurately assessing the value of mindfulness to work. In applied studies examining the effect of mindfulness practices and interventions, various antecedents may generate substantial noise confounding results. Null effects from an intervention may therefore arise from individuals practicing in distracting environments or feeling unconfident employing mindfulness practices at work, rather than arising from the psychological quality of mindfulness itself.

#### Outcomes of mindfulness at work

Our findings aligned with previously-identified outcomes from mindfulness at work (Good et al., 2016), while clarifying these impacts may arise through the interaction between Being and Doing. This new contribution emerged as informants described how Doing mode properties could be liabilities that yielded undesirable workplace outcomes. Complete reliance on Doing mode properties that typically guide workplace functioning can be analogized to a toolbox with limited tools. This makes some problems readily fixable, but makes addressing other issues difficult or impossible. For example, when managing a subordinate who erred, Doing mode might elicit harsh responses that could undermine effective correction of the error and subordinate well-being. In such instances, Doing mode was like using a screwdriver to hammer a nail.

Conversely, when Disentangled, individuals managing a situation could draw upon the broader repertoire of cognitive properties added by Being mode. In this instance, having some acceptance of the mistake could allow for maintaining the relationship with the subordinate while still permitting calmer error detection and correction. By allowing for a more complex cognitive response, mindfulness may facilitate more effective management of the situation and better outcomes. Being mode seemingly provided the right tools across a wider array of situations, offering an expanded array of cognitive resources to optimize workplace functioning. We extend these ideas with respect to MOC literature in the following section.

#### Interrelating mindfulness with managerial and organizational cognition (MOC)

What impacts and value does mindfulness have for work? Because our results suggest that mindfulness provides a set of alternative if not opposite cognitive properties for engaging work, this may explain much of mindfulness' broad and fundamental influence over workplace functioning (Good et al., 2016).

These changes are sufficiently dramatic that mindfulness may present a significant boundary condition on existing theories of MOC. These generally assume that core aspects of Doing mode govern workplace functioning (e.g., Walsh, 1995; Lord et al., 2010), a tenuous assumption when Being mode enables a very different set of cognitive properties (see Tables 1, 4). Mindful individuals may therefore function quite differently than predicted by MOC theories. Being mode properties may act as resources enabling alternative psychological processes and outcomes, suggesting existing models may need revision to explain the behavior of mindful individuals at work. For example, such workers may not evaluate events as good or bad, instead dropping this evaluative appraisal and accepting an event's existence. The implications of mindfulness are potentially vast and well beyond the scope of this paper to fully consider, so we instead suggest general guidance for future theorizing.

The workplace impacts of mindfulness appear fundamentally influenced by the relationship between Being and Doing. Disentangled individuals reported that the cognitive properties of Being mode could replace or activate alongside Doing mode properties. These individuals reported having sets of properties from both modes available to shape a psychological process (e.g., self-regulation). Sometimes Being mode properties substituted for comparable Doing mode properties (e.g., acceptance instead of evaluation), while in other instances both properties were co-active (e.g., simultaneous acceptance and evaluation). Rather than being locked into judgmental evaluation or acceptance of a situation, a Disentangled manager may actually have both cognitive properties ongoing simultaneously.

Our data suggest that despite fears about potential negative effects (Dane, 2011), mindfulness may be typically benign for work. The rationale for this principle is that Disentanglement may allow mindfulness to serve as a resource for workplace functioning without becoming a liability. If Being and Doing are Incompatible, then mindfulness may imply cognitive trade-offs when its properties undermine functioning. In such cases, it would be a liability; a manager who could only accept but not evaluate strategic options would likely be ineffective. Disentangled individuals did not report suffering from this limitation, as they reported activating and employing properties from both modes, seemingly allowing for the qualities of Being mode to govern their functioning when these were helpful. In such individuals, mindfulness apparently served as a resource supporting workplace functioning without restricting use of Doing mode properties that were adaptive.

Because mindfulness significantly disrupts prototypical cognitive functioning, scholars should consider how MOC theories reliant upon Doing mode properties transfer to mindful individuals. As mindful individuals may think and act in ways unexplained by these models, mindfulness may then present a boundary condition for theories such as self-regulation (Carver and Scheier, 1982; Lord et al., 2010), decision-making and interpretation (March and Simon, 1958; Weick, 1979; Walsh, 1995), core self-evaluations (Judge et al., 2002), and identity (Ashforth et al., 2008; Brockner and Wiesenfeld, 2016). Future MOC theorists may benefit from considering how the relationship and properties of Being and Doing modes found in Tables 1, 4 shape these psychological processes.

The properties of Being mode may then intervene and substantively alter a given psychological process. We demonstrate this approach by reconsidering how Being mode may intersect Carver and Scheier's (1982) theory of cybernetic self-regulation. Often invoked in MOC discourse (e.g., Lord et al., 2010), we chose this model for its explicit reliance on Doing mode properties^4^ This discrepancy-reduction model presumes that individuals can predict the value of future realities for their self in relation to goals. This cognitive process then automatically guides behavior in the present. A Disentangled individual may employ an alternative self-regulation model, as informants reported several cognitive processes that were different than assumed in cybernetic theory (see Tables 1, 4). For example, they did not primarily anchor their perception in the future or their self, and their appraisals of situations as good or bad often co-occurred with a more fundamental sense of acceptance. Although mindfulness supports effective self-regulation at work and beyond (Brown et al., 2007; Glomb et al., 2011; Good et al., 2016), our data shows this occurs despite maintaining cognitive properties that could undermine cybernetic self-regulation. How remains unclear, and presents an opportunity for retheorizing self-regulation in mindful individuals. A similar approach may be fruitful with other MOC theories.

#### Future research directions

##### Advancing the being and doing relationship

This nascent view of mindfulness at work raises a number of important investigations for future research. While we identify new constructs of Disentanglement and Entanglement, subsequent research may attempt to validate and refine these constructs through qualitative, quantitative, and neurophenomenological approaches. Further description of the experience of mindfulness at work in a context with antecedents of Entanglement (e.g., events provoking intense negative emotions) might advance our initial conception. Developing quantitative assessments of Being or Doing would also be beneficial. This might include assessing if reported facets of experiences (e.g., automatic thoughts or mental quiet) load onto latent factors reasonably construed as Entanglement or Disentanglement, and whether such constructs are linked to antecedents (e.g., facing incivility) and outcomes (e.g., stress) as described in qualitative reports. Finally, linking experiential reports of mindfulness with neural activity while engaging workplace situations like interpersonal interactions or task performance could robustly triangulate our findings. Consistent with our model, one such study dichotomized phenomenological reports during meditation into experiences of being either “controlling” or “effortless,” and these corresponded with activity levels in a brain region linked to belief in thoughts and self-reference (Garrison et al., 2013). Similar efforts linking such experiences and neural correlates to workplace functioning would be a logical next step.

##### Mindfulness self-efficacy and competencies

Future research should also systematically address variation in individuals' perceived and enacted ability to be mindful while doing work. In our data, we identified *practical mindfulness self-efficacy* as the self-perceived ability to perform mindfulness practices at work. This impacted the frequency of mindfulness practice and therefore transitions. We should note that our conception of mindfulness self-efficacy differs from previous usage found in Chang et al. (2004). They initially linked this concept to mindfulness, but diverged from Bandura's (1977a) definition of self-efficacy beliefs as self-appraised likelihood of performing a behavior successfully. Instead they define “mindfulness self-efficacy” as the capability to be consistently mindful. In our view this actually reflects a competency (Boyatzis, 1982), which also seems likely to explain the outcomes of dispositional mindfulness and mindfulness practices.

Therefore, we envision future research on both *workplace mindfulness self-efficacy* and *competencies*. This might involve assessing *practical self-efficacy* and *competency* around performing mindfulness practices at work. It may also involve *cognitive mindfulness self-efficacy*, the self-perceived ability to maintain a Continuity of mindfulness while working. Finally, researchers might evaluate *cognitive mindfulness competency* to maintain this Continuity.

We imagine these four constructs as being vital to understanding mindfulness at work. These may help to clarify variations in the ability to be mindful while doing work, a potentially significant confound. Working individuals who maintain a Continuity of mindfulness across circumstances should derive more consistent benefit from mindfulness than those experiencing frequent Entanglement, variability not easily assessed today. Future research in this area might include qualitative investigations of the factors and processes influencing Disentanglement and Continuity of mindfulness in workplace contexts. These findings might inform an assessment of mindfulness self-efficacy and competency at work.

##### Mindfulness at work as a potential paradox

Theories of mindfulness at work may benefit from the paradox lens. Being Disentangled might be viewed as effectively managing paradoxical tensions within one's mind emerging at the nexus of Being and Doing. The emerging paradox literature emphasizes the value of managing tensions between mutually incompatible and interdependent elements (Smith and Lewis, 2011; Lewis and Smith, 2014). Little is known about how to transcend such tensions, particularly within individuals (Jules and Good, 2014). This appears challenging, which may explain why Disentanglement is unstable and must be continuously achieved. Future research should explore whether Being and Doing modes reflect a paradox that individuals transcend with varying competency, and how managing these tensions influences organizational processes and outcomes.

## Practical implications

We label the robust and growing movement to integrate mindfulness into the workplace (Good et al., 2016) as the emerging discipline of *contemplative management*. Our findings show that integrating mindfulness at work fundamentally involves balancing Being and Doing modes. Therefore, we view the primary activity of contemplative management as enabling “Being while Doing” to support individual and organizational functioning.

Contemplative management currently lacks a framework to systematically guide management of Being while Doing. We begin to lay the theoretical groundwork for this practice through our model of mindfulness at work in Figure 2. Despite deployment within a context demanding continuous Doing mode, current workplace training approaches typically apply principles for practice developed in contemplative contexts like meditation retreats devoted to Being. By systematically interrelating Being and Doing, the framework may therefore help guide effective mindfulness practice in this new and different environment.

This study surfaced two concepts particularly relevant to contemplative management practice: transitions between Entanglement and Disentanglement, and antecedent factors impacting these transitions. We identify sustained Disentanglement—e.g., a Continuity of mindfulness—as an ideal experience of mindfulness at work. Avoiding the transition to Entanglement allowed individuals to derive sustained benefits from mindfulness. Training should therefore emphasize the recognition and management of these transitions. Individuals must develop skills not only in being mindful, but also in noting when they are unmindful and skillfully return back to mindfulness when appropriate. While unclear in our data, individuals who successfully maintain Continuity may quickly detect and halt the process of Entanglement, so trainers ideally would offer practices effective in achieving Disentanglement.

Practitioners must also be aware of how situational, behavioral, and individual factors impact how they manage these transitions. Situational features both supported and undermined our informants' ability to be mindful at work. Trainers should therefore help individuals identify, select, and manage situations to facilitate being mindful when this is desired. For example, training might support individuals in conducting mindfulness practices not just in ideal conditions, but also in demanding real-world situations. This may help augment individuals' practical self-efficacy, and support them in practicing with sufficient frequency to maintain a Continuity of mindfulness.

In closing, contemplative management supports individuals in managing the challenges with being mindful at work. We hope this study provides an initial basis for developing evidence-based practices that facilitate Being *despite* Doing.

## Study limitations

As a broad exploratory study intended to map the experience of mindfulness at work, the study is intrinsically limited in its specificity and depth in any given area of inquiry. The limited sample size may have constrained reports to a narrow cross-section of the overall population. The population selected for interviews may not be representative of mindful professionals, in terms of mindfulness experience or practices, or professional roles. While the study design was intended to minimize this issue by gathering a heterogeneous cross-section of individuals interested in being mindful at work, this bias cannot be ruled out.

Our focus on the experience of mindfulness at work may have left gaps regarding the antecedents and outcomes. We documented some antecedent factors of the Being-Doing relationship. However, we do not purport to have an exhaustive list, there may well be other factors that impact this relationship. These features also seem likely to interact (e.g., attentional demands may interfere with performing meditation successfully among individuals with low practical mindfulness self-efficacy), but we were unable to systematically document these potentially important connections.

## Conclusion

Organizational science has developed by integrating new ideas from psychology. Mindfulness presents a significant shift in the human mind that is only now being connected to work. Clinical psychologists have described three waves of therapeutic approaches: behavioral interventions into stimulus-response links, cognitive-behavioral interventions into the content of thoughts, and mindfulness-inspired techniques that alter individuals' relationship to thoughts (Kahl et al., 2012). The cognitive-behavioral transition initiated the current paradigm in MOC (e.g., Bandura, 1977b; Weick, 1979). While far too early to predict a similar contribution, we see Being mode as offering a similarly radical reconception of the human mind. At a minimum, managing Being mode anchors the emerging discipline of *contemplative management*.

Mindfulness at work may seem challenging, but our findings suggest this is a manageable and valuable combination. Organizational theorists have long emphasized the dominance and value of Doing mode. Yet mindfulness theory and practice show the limitations of this lens. Williams and Penman (2012, p. 36) suggest that in fact, “Doing mode … becomes a ‘problem’ when it volunteers for a task that it cannot do. … When this happens, it pays to ‘shift gear’ into ‘Being’ mode. This is what mindfulness gives us: the ability to shift gears as we need to, rather than being permanently stuck in the same one.” Our findings regarding Entanglement and Disentanglement illustrate the nature and impacts of transitioning from Doing alone, to Being while Doing.

## Ethics statement

Data collection was approved by the Case Western Reserve University Institutional Review Board. Participants were asked to complete semi-structured interviews ranging from 30 to 150 min regarding their experience of mindfulness in the workplace. They were asked to sign paper or digital forms indicating consent to participate in the interview, which included information about the potential risks and benefits of participating in the study. Some participants were organizational members and were deemed vulnerable populations. These were invited to participate through organizational contacts. In these cases, interviews were conducted by CL in private locations secluded from any organizational members who had involvement in mindfulness training or research that may have comprised part of the interviews.

## Author contributions

CL developed the research questions and methodology, and conducted interviews. CL and DG co-analyzed the data and co-authored the manuscript.

## Funding

Darren Good gratefully acknowledges support from the Julian Virtue Professorship endowment.

### Conflict of interest statement

The authors declare that the research was conducted in the absence of any commercial or financial relationships that could be construed as a potential conflict of interest.

## Footnotes

^1^This definition contrasts with standing conceptions as cognitive flexibility and reliable collective performance (Langer, 1989; Weick et al., 1999).

^2^In this article we equate *Being* mode with mindfulness and *Doing* mode with the goal-oriented mode of operating typically thought to undergird work.

^3^Many vignettes were lengthy, with some exceeding 2,000 words, necessitating presentation of edited vignettes for space considerations.

^4^The discrepancy-reduction processing of cybernetic self-regulation is equivalent to Doing mode (Williams, 2008).
